# Supplementary material for: Pyruvate Dehydrogenase Kinase 1 inhibition mediated oxidative phosphorylation enhancement in cartilage promotes osteoarthritis progression
Source: BMC Musculoskelet Disord. 2023 Jul 20;24:597. doi: 10.1186/s12891-023-06585-6 (PMC10357736; doi:10.1186/s12891-023-06585-6)
Supplement: Supplementary file 1 — Additional file 1. [file 12891_2023_6585_MOESM1_ESM.zip › KEGG.Gsea.1653622432464/KEGG_MISMATCH_REPAIR.html]

Details for gene set KEGG\_MISMATCH\_REPAIR[GSEA]

|  || Dataset | 55457\_collapsed\_to\_symbols.cls.cls #OA\_versus\_normal.cls.cls #OA\_versus\_normal\_repos |
| Phenotype | cls.cls#OA\_versus\_normal\_repos |
| Upregulated in class | OA |
| GeneSet | KEGG\_MISMATCH\_REPAIR |
| Enrichment Score (ES) | 0.63443744 |
| Normalized Enrichment Score (NES) | 2.1440094 |
| Nominal p-value | 0.0 |
| FDR q-value | 8.2497625E-4 |
| FWER p-Value | 0.002 |
Table: GSEA Results Summary

  

Fig 1: Enrichment plot: KEGG\_MISMATCH\_REPAIR      
 Profile of the Running ES Score & Positions of GeneSet Members on the Rank Ordered List

  

| SYMBOL | TITLE | RANK IN GENE LIST | RANK METRIC SCORE | RUNNING ES | CORE ENRICHMENT || 1 | RPA3 | replication protein A3 [Source:HGNC Symbol;Acc:HGNC:10291] | 13 | 1.159 | 0.1158 | Yes |
| 2 | RPA2 | replication protein A2 [Source:HGNC Symbol;Acc:HGNC:10290] | 339 | 0.727 | 0.1639 | Yes |
| 3 | MLH1 | mutL homolog 1 [Source:HGNC Symbol;Acc:HGNC:7127] | 497 | 0.660 | 0.2182 | Yes |
| 4 | RFC3 | replication factor C subunit 3 [Source:HGNC Symbol;Acc:HGNC:9971] | 570 | 0.633 | 0.2763 | Yes |
| 5 | RPA1 | replication protein A1 [Source:HGNC Symbol;Acc:HGNC:10289] | 571 | 0.632 | 0.3400 | Yes |
| 6 | MSH2 | mutS homolog 2 [Source:HGNC Symbol;Acc:HGNC:7325] | 739 | 0.584 | 0.3859 | Yes |
| 7 | POLD1 | "DNA polymerase delta 1, catalytic subunit [Source:HGNC Symbol;Acc:HGNC:9175]" | 811 | 0.566 | 0.4375 | Yes |
| 8 | POLD4 | "DNA polymerase delta 4, accessory subunit [Source:HGNC Symbol;Acc:HGNC:14106]" | 820 | 0.564 | 0.4937 | Yes |
| 9 | RFC4 | replication factor C subunit 4 [Source:HGNC Symbol;Acc:HGNC:9972] | 897 | 0.543 | 0.5426 | Yes |
| 10 | RFC1 | replication factor C subunit 1 [Source:HGNC Symbol;Acc:HGNC:9969] | 931 | 0.531 | 0.5936 | Yes |
| 11 | RFC2 | replication factor C subunit 2 [Source:HGNC Symbol;Acc:HGNC:9970] | 1247 | 0.467 | 0.6162 | Yes |
| 12 | PCNA | proliferating cell nuclear antigen [Source:HGNC Symbol;Acc:HGNC:8729] | 1557 | 0.419 | 0.6344 | Yes |
| 13 | SSBP1 | single stranded DNA binding protein 1 [Source:HGNC Symbol;Acc:HGNC:11317] | 3038 | 0.246 | 0.5443 | No |
| 14 | MLH3 | mutL homolog 3 [Source:HGNC Symbol;Acc:HGNC:7128] | 4335 | 0.125 | 0.4563 | No |
| 15 | POLD2 | "DNA polymerase delta 2, accessory subunit [Source:HGNC Symbol;Acc:HGNC:9176]" | 6311 | -0.028 | 0.3058 | No |
| 16 | LIG1 | DNA ligase 1 [Source:HGNC Symbol;Acc:HGNC:6598] | 6944 | -0.075 | 0.2643 | No |
| 17 | POLD3 | "DNA polymerase delta 3, accessory subunit [Source:HGNC Symbol;Acc:HGNC:20932]" | 7568 | -0.125 | 0.2285 | No |
| 18 | EXO1 | exonuclease 1 [Source:HGNC Symbol;Acc:HGNC:3511] | 8088 | -0.162 | 0.2046 | No |
| 19 | PMS2 | "PMS1 homolog 2, mismatch repair system component [Source:HGNC Symbol;Acc:HGNC:9122]" | 8172 | -0.169 | 0.2151 | No |
| 20 | MSH6 | mutS homolog 6 [Source:HGNC Symbol;Acc:HGNC:7329] | 8846 | -0.221 | 0.1851 | No |
| 21 | RFC5 | replication factor C subunit 5 [Source:HGNC Symbol;Acc:HGNC:9973] | 9499 | -0.274 | 0.1621 | No |
| 22 | MSH3 | mutS homolog 3 [Source:HGNC Symbol;Acc:HGNC:7326] | 9796 | -0.301 | 0.1695 | No |
| 23 | RPA4 | replication protein A4 [Source:HGNC Symbol;Acc:HGNC:30305] | 12406 | -0.709 | 0.0384 | No |
Table: GSEA details [plain text format]

  

Fig 2: KEGG\_MISMATCH\_REPAIR      
 Blue-Pink O' Gram in the Space of the Analyzed GeneSet

  

Fig 3: KEGG\_MISMATCH\_REPAIR: Random ES distribution      
 Gene set null distribution of ES for **KEGG\_MISMATCH\_REPAIR**
